# The Hidden Agony of the Pancreas: A Comprehensive Case Study of Paraduodenal Pancreatitis

**DOI:** 10.7759/cureus.63149

**Published:** 2024-06-25

**Authors:** Guangbin Chen, Yanguang Sha, Lifang Chen, Dingbang Wang, Rongmei Tang

**Affiliations:** 1 Department of Hepatobiliary Surgery, The Second People’s Hospital of Wuhu, Wuhu Hospital Affiliated to East China Normal University, Wuhu, CHN; 2 Graduate School, Wannan Medical College, Wuhu, CHN; 3 Department of Medical Imaging, The Second People’s Hospital of Wuhu, Wuhu Hospital Affiliated to East China Normal University, Wuhu, CHN

**Keywords:** step-up approach, diagnostic imaging, chronic pancreatitis, groove pancreatitis, paraduodenal pancreatitis

## Abstract

Paraduodenal pancreatitis (PP), also known as groove pancreatitis (GP), is a rare and distinct variant of chronic pancreatitis and presents significant diagnostic and therapeutic challenges. This comprehensive case study explores a 54-year-old male patient’s journey, highlighting the intricate relationship between clinical presentation, diagnostic modalities, and management strategies. Despite a history of smoking and alcohol consumption, the diagnosis of PP was primarily reliant on advanced imaging techniques, including computed tomography and magnetic resonance imaging, which revealed characteristic findings of GP. The case underscores the importance of a high index of suspicion and a step-up approach to management, starting with conservative treatment and progressing to surgical intervention as necessary. This study contributes to the growing body of knowledge on PP, emphasizing the need for awareness and understanding of this rare condition to improve patient outcomes.

## Introduction

Paraduodenal pancreatitis (PP) is a unique and rare variant of chronic pancreatitis that presents significant diagnostic and therapeutic challenges [1]. First described in 1982, PP is characterized by inflammation and fibrosis in the anatomical region between the pancreatic head, duodenum, and common bile duct, known as the “groove” area [2]. This condition predominantly affects middle-aged men, particularly those with a history of chronic alcohol consumption and smoking, which are significant risk factors for the development of PP [3,4].

The diagnosis of PP heavily relies on advanced imaging techniques, particularly computed tomography (CT) and magnetic resonance imaging (MRI), which are crucial for identifying the characteristic findings associated with this condition [1,5]. Management of PP follows a “step-up” approach, starting with conservative treatment, including pain relief and alcohol cessation, and progressing to endoscopic and surgical interventions as necessary [6].

This comprehensive case study explores the journey of a 54-year-old male patient diagnosed with PP, highlighting the intricate relationship between clinical presentation, diagnostic modalities, and management strategies. By detailing this case, we aim to contribute to the growing body of knowledge on PP, emphasizing the need for awareness and understanding of this rare condition to improve patient outcomes.

## Case presentation

A 54-year-old male patient was admitted to the hospital due to discomfort in the upper right abdomen after eating, which had persisted for seven days. This discomfort was accompanied by continuous radiating back pain, with symptoms worsening at night. Despite receiving symptomatic treatment such as intravenous fluids and pain relief at a local clinic, there was no significant improvement. The patient sought further diagnosis and treatment at our hospital. There were no notable instances of jaundice in the skin or sclera during the illness. The patient had a history of smoking for 30 years and drinking alcohol for 20 years. Family history included his mother dying from gallbladder cancer, his father from a gastrointestinal tumor, and his brother being diagnosed with pancreatic cancer last year.

Upon admission, comprehensive auxiliary examinations were conducted. The laboratory investigations are summarized in Table 1.

**Table 1:** Laboratory investigations upon admission.

Parameter	Result	Reference range	Interpretation
White blood cells	11.53 × 10^9^/L	4.0–11.0 × 10^9^/L	Increased
Neutrophil percentage	66.20%	40%–70%	Normal
Alanine aminotransferase	24 U/L	7–56 U/L	Normal
Aspartate aminotransferase	23 U/L	10–40 U/L	Normal
Gamma-glutamyltransferase	27 U/L	9–48 U/L	Normal
Alkaline phosphatase	75 U/L	44–147 U/L	Normal
Total bilirubin	12.8 µmol/L	5.1–17.1 µmol/L	Normal
Direct bilirubin	3.0 µmol/L	0–6.8 µmol/L	Normal
Indirect bilirubin	9.8 µmol/L	3.4–13.7 µmol/L	Normal
Amylase	68 U/L	30–110 U/L	Normal
Alpha-fetoprotein	6.39 ng/mL	<10 ng/mL	Normal
Carcinoembryonic antigen	0.7 ng/mL	<5 ng/mL	Normal
Carbohydrate antigen 125	15.4 U/mL	<35 U/mL	Normal
Carbohydrate antigen 19-9	1.7 U/mL	< 37 U/mL	Normal

**Figure 1:**
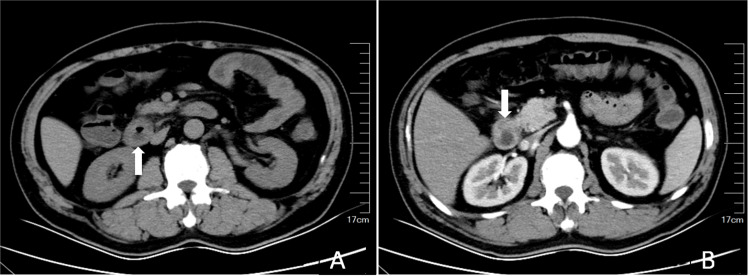
Contrast-enhanced CT scan of the upper abdomen: plain phase (A) and arterial phase (B). CT scan showed signs of thickening of the descending part of the duodenal wall (white arrow). CT: computed tomography

Upper abdominal CT scans, both plain and enhanced, indicated signs of chronic cholecystitis and possible small gallstones, suggesting groove pancreatitis (GP) (Figure 1).

**Figure 2:**
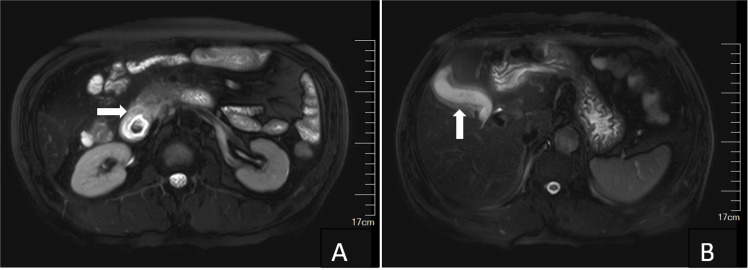
Axial-unenhanced MRI of the patient’s abdomen. T2-weighted image demonstrated slight effusion around the head of the pancreas and thickening of the descending part of the duodenum (A, white arrow), signs of chronic cholecystitis, and sludge-like gallstones (B, white arrow). MRI: magnetic resonance imaging

MRI showed slight effusion around the head of the pancreas and slight thickening of the descending part of the duodenum, with GP pending further evaluation, signs of chronic cholecystitis, and sludge-like gallstones (Figure 2).

After admission, the patient received anti-inflammatory treatment, acid suppression therapy, and parenteral nutrition support. The patient’s symptoms significantly improved, leading to discharge with plans for an elective cholecystectomy.

## Discussion

PP, also known as GP, is a rare and distinct variant of chronic pancreatitis that primarily affects the anatomical region between the pancreatic head, duodenum, and common bile duct, referred to as the “groove” area [4]. The term “groove pancreatitis” was first introduced by Stolte et al. in 1982 following their analysis of specimens from 30 patients, which led to the delineation of its unique characteristics [2]. In 2004, Adsay and colleagues further elucidated that previously considered separate duodenal lesions shared common pathological features, warranting their collective classification under the umbrella of PP [4].

The diagnosis of PP heavily relies on radiological evidence, particularly through the use of CT and MRI [1,3,7,8]. These advanced imaging modalities play a crucial role in identifying the characteristic findings associated with PP, such as thickening of the duodenal wall, cystic changes in the duodenal wall, and inflammatory changes in the pancreaticoduodenal groove [1,5,8]. In the presented case, both CT and MRI findings were suggestive of GP, highlighting the importance of these diagnostic tools in the evaluation of PP.

PP is most commonly observed in middle-aged men, particularly between the ages of 40 and 50 [9,10]. Although the exact etiology of PP remains unclear, it is believed to have a multifactorial origin. Chronic excessive alcohol consumption has been identified as a significant risk factor, as it can increase the viscosity of pancreatic secretions and induce calcification within the pancreatic ducts, thereby impeding the normal flow of pancreatic fluids [3]. Moreover, smoking has been shown to increase the viscosity of pancreatic secretions and promote calcification within the pancreatic ducts, further impeding the normal flow of pancreatic fluids [11]. In the current case, the middle-aged patient’s long-standing history of alcohol consumption and cigarette smoking likely played a significant role in the development of PP, underscoring the importance of addressing this modifiable risk factor in the management of the disease.

The management of PP follows a “step-up” approach, starting with conservative treatment and progressing to endoscopic and surgical interventions as necessary [6,12,13]. Conservative management, including pain relief and alcohol cessation, is considered appropriate for patients in the early stages of the disease [6]. Endoscopic treatments, such as dilation of the minor papilla, can be employed to restore adequate drainage of pancreatic juice in cases of impaired drainage [12]. Surgical interventions, including standard pancreaticoduodenectomy and pylorus-preserving pancreaticoduodenectomy, are reserved for advanced cases that do not respond to conservative or endoscopic treatment [6,13].

## Conclusions

PP presents significant diagnostic and therapeutic challenges due to its rarity and complex clinical presentation. This case study highlights the importance of maintaining a high index of suspicion for PP, especially in middle-aged men with a history of chronic alcohol consumption and smoking. Advanced imaging modalities such as CT and MRI are essential tools for accurate diagnosis, revealing characteristic findings that guide clinical management. The step-up approach to treatment, beginning with conservative measures and progressing to endoscopic and surgical interventions as necessary, underscores the need for individualized patient care. Future research should focus on further elucidating the pathogenesis of PP and identifying novel strategies to improve patient outcomes.
